# NOD1 induces pyroptotic cell death to aggravate liver ischemia‐reperfusion injury in mice

**DOI:** 10.1002/mco2.170

**Published:** 2022-08-31

**Authors:** Yu Liu, Shipeng Li, Guoliang Zhang, Jinzhen Cai

**Affiliations:** ^1^ Department of Gastroenterology Tianjin First Central Hospital The First Central Clinical College Tianjin Medical University Tianjin China; ^2^ Department of internal medicine Wangdingdi Hospital Nankai District Tianjin China; ^3^ Department of General Surgery Jiaozuo People's Hospital Xinxiang Medical University Jiaozuo China; ^4^ Department of organ transplantation Organ Transplant Center Affiliated Hospital of Qingdao University Qingdao China

**Keywords:** hepatocytes, ischemia‐reperfusion, NOD1, pyroptosis

## Abstract

Nucleotide‐binding oligomerization domain 1 (NOD1) can direct the release of inflammatory factors and influence autophagy and apoptosis in hepatic ischemia‐reperfusion injury (IRI) in mice. As pyroptosis is involved in a number of inflammatory reactions, in this report, we investigated the potential for NOD1 to affect pyroptosis. We found that an increased expression of NOD1 during IRI was related to activation of the pyroptotic signaling pathway. With NOD1 activation, cleavage fragments of Caspase‐1, gasdermin D (GSDMD), and interleukin (IL)‐1β were all increased. Moreover, downregulation of NOD1 expression in AML12 cells exerted an opposite effect. Expression levels of cleaved‐Caspase‐1 and cleaved‐GSDMD decreased after exposure to IRI and the number of cell membrane pores and apoptotic or pyroptotic cells decreased, along with the contents of inflammatory factors and lactate dehydrogenase in the supernatants of AML12 cells. Based on these findings, we conclude that NOD1 aggravates the pyroptotic cell death associated with hepatic ischemia‐reperfusion injury in a mouse model via the Caspase‐1/GSDMD axis. These findings help to alleviate pyroptotic cell death during liver transplantation or resection, providing new insights into novel protective therapies for liver IRI.

## Graphical abstract

NOD1 is involved in apoptosis and autophagy during hepatic ischemia‐reperfusion. In this paper, we investigated its relationship with pyroptosis. The results showed that activation of NOD1 significantly aggravated pyroptotic cell death, resulting in liver injury, which has positive significance for further research on how to reduce liver ischemia‐reperfusion injury during liver transplantation or resection by affecting NOD1.

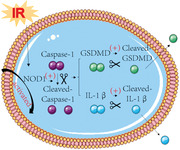

## 1 INTRODUCTION

Liver ischemia‐reperfusion injury (IRI), which represents a significant basis for liver injury and graft dysfunction, often results from processes involved in liver transplantation or resection.^1^ Therefore, an understanding of specific mechanisms involved with liver IRI has the potential to reduce the complications of liver resection and transplantation and thus improve the prognosis of these patients.^2^ IRI is a two‐stage process with the initial decrease in blood flow to an organ leading to hypoxia and resultant cell damage, effects that are then worsened after restoration of oxygen delivery.^1^ Liver IRI involves an array of events consisting of anaerobic metabolism, mitochondrial dysfunction, oxidative stress, innate immune responses, and activation of inflammatory factors. The excessive degree of injury resulting from IRI leads to apoptosis and necrosis of liver parenchymal cells.^3^, ^4^

An additional factor that has been found to play a critical role in liver IRI is that of pyroptosis. This recently identified type of programmed death, which is distinct from that of apoptosis and necrosis,^5^ involves an activation of innate immune mechanisms against pathogen infection. Classical pathway requires that NOD proteins in Nod‐like receptors (NLRs), an inflammasome composed of Nalp3 (Nlrp3), activate the key protease Caspase‐1 precursor. The normally dormant Caspase‐1 precursor is self‐activated by proteolytic cleavage into active heterodimers composed of 10 and 20 kDa chains.^6^, ^7^ This newly activated caspase‐1 simultaneously induces the cleavage of IL‐1β and IL‐18 precursors, while also cleaving GSDMD into C‐ and N‐terminal fragments. The N‐terminal fragment aggregates onto cell membrane to form pores, and the cleaved IL‐1β and IL‐18 products are released from the pore.^8^, ^9^, ^10^ With pyroptosis, cell swelling, membrane pore formation, rapid lysis, and release of proinflammatory mediators represent the significant components involved with this programmed cell death.^11^ While immune responses against pathogens contribute to cellular self‐protection, excessive pyroptosis leads to cell death.^12^

A related factor involved with the apoptosis and autophagy of liver IRI is that of nucleotide‐binding oligomerization domain 1 (NOD1). NOD1 is not only associated with inflammatory diseases, but also immune responses.^13^ NOD1 consists of C‐terminal leucine‐rich repeats, a central NACHT domain, and an N‐terminal caspase recruitment domain (CARD).^14^ As CARD is highly conserved in NOD1, this CARD can recruit caspases, the key mediator of apoptosis and pyroptosis.^11^, ^15^ In this way, NOD1 can recognize peptidoglycan through N‐terminal caspase recruitment and activation of the CARD domain to activate the NF‐KB pathway through interactions with adapter kinase RIP2,^16^ leading to the expression of pro‐IL‐18^17^, ^18^ and pro‐IL‐1β.^18^, ^19^ Activation of NOD1 has also been shown to be involved with PI3K/Akt and MAPK pathways, promotion of cell apoptosis and inflammatory responses.^20^

Given the above information, we considered the possibility that NOD1 may participate in cellular pyroptosis. Therefore, in this study we examined the impact and some of the underlying mechanisms of NOD1 in pyroptosis as assessed in both in vitro and in vivo models of hepatic IRI. These findings can serve as a basis for the development of new targets directed at pyroptosis, which have the potential of modulating liver IRI.

## 2 RESULTS

### 2.1 Serum levels of ALT, AST, IL‐1β, and IL‐18 along with pathological and morphological alterations in a mouse model of liver IRI

**FIGURE 1:**
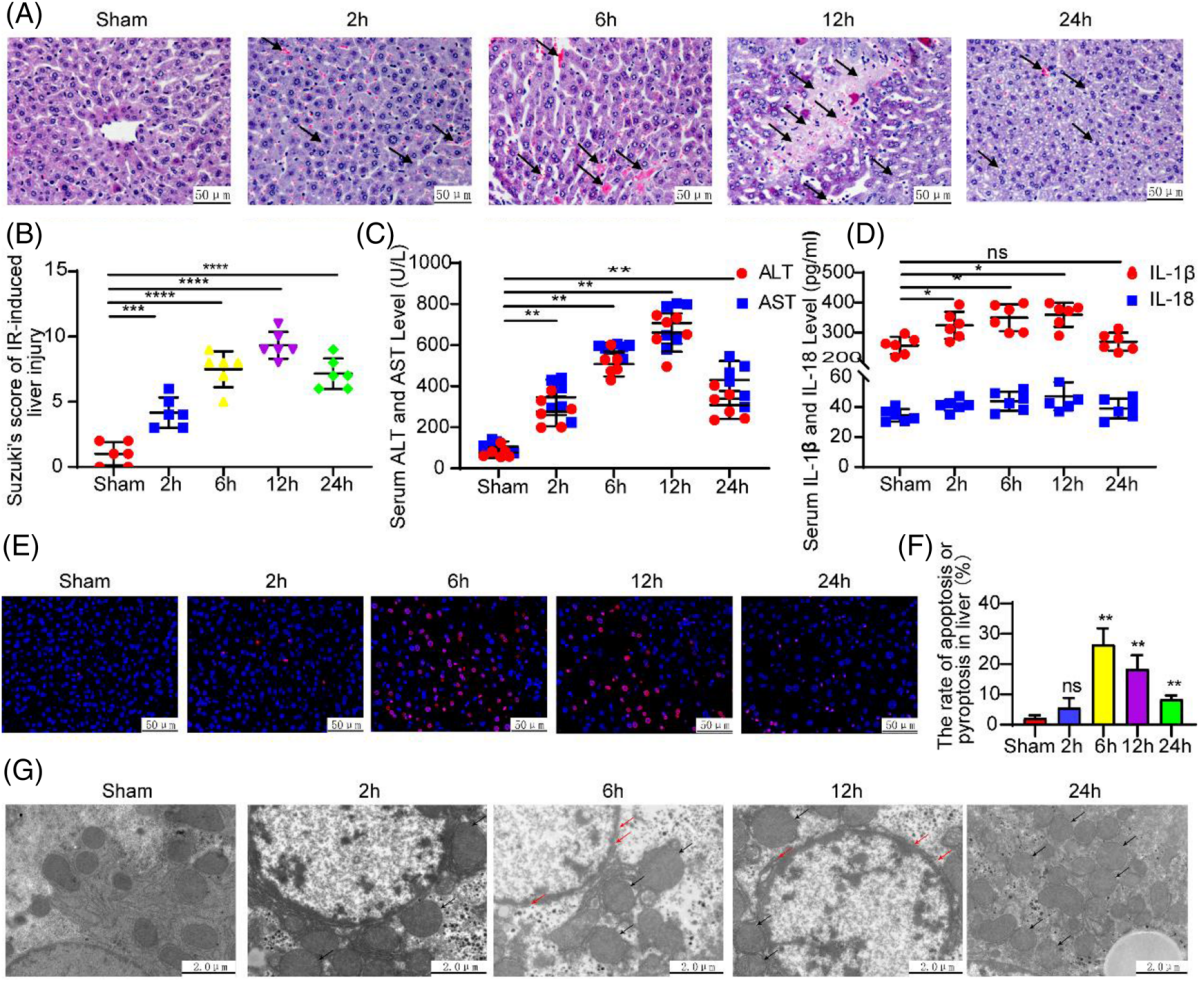
Serology, pathology, and morphology in a mouse hepatic IRI model. (A) Pathological changes in an IR liver (×200, black arrow). (B) Suzuki's score. (C) IR increased serum ALT and AST, with levels reaching a peak at approximately 12 h. (D) Serum contents of IL‐1β and IL‐18 in the IR group were increased as compared with those in the sham group. (E and F) Cell apoptosis or pyroptosis as measured using TUNEL and analysis of TUNEL‐positive cells (apoptotic nuclei were stained red and normal nuclei blue). (G) TEM images of mouse hepatocytes after IR. Compared with sham group: *p < 0.05, **p < 0.01, ***p < 0.001, ****p < 0.0001

Results from histological examinations following hepatic ischemia‐reperfusion (IR) revealed an aggravation of hepatic sinusoid congestion and the presence of extensive hepatocyte necrosis and vacuolations. These effects were gradually enhanced over the following 2–12 h period post‐IR, and then the injury decreased (Figures 1A and 1B; *p* < 0.001). Serum levels of ALT and AST gradually increased over the reperfusion time, achieving peak levels at 12 h and decreasing thereafter as indicated from enzyme‐linked immunosorbent assay (ELISA) results (Figure 1C; *p* < 0.01). Similarly, serum IL‐1β levels were significantly increased after IR, while only slight increases were observed in IL‐18 levels (Figure 1D; IR2, 6, 12 h vs. Sham; *p* < 0.05). TUNEL assay results indicated that apoptotic and pyroptotic cell numbers gradually increased in the IR group reaching peak levels at 6 h, followed by a gradual decrease, with these changes being significantly increased over that in the sham group (Figures 1E and 1F; IR6, 12, 24 h vs. Sham; *p* < 0.01). We also found that pore formations within hepatocyte or nuclear membranes in the IR group were increased along with an increased degree of cell damage as shown with transmission electron microscopy (TEM) (Figure 1G).

### 2.2 NOD1 and pyroptosis indicators in a mouse model of liver IRI

**FIGURE 2:**
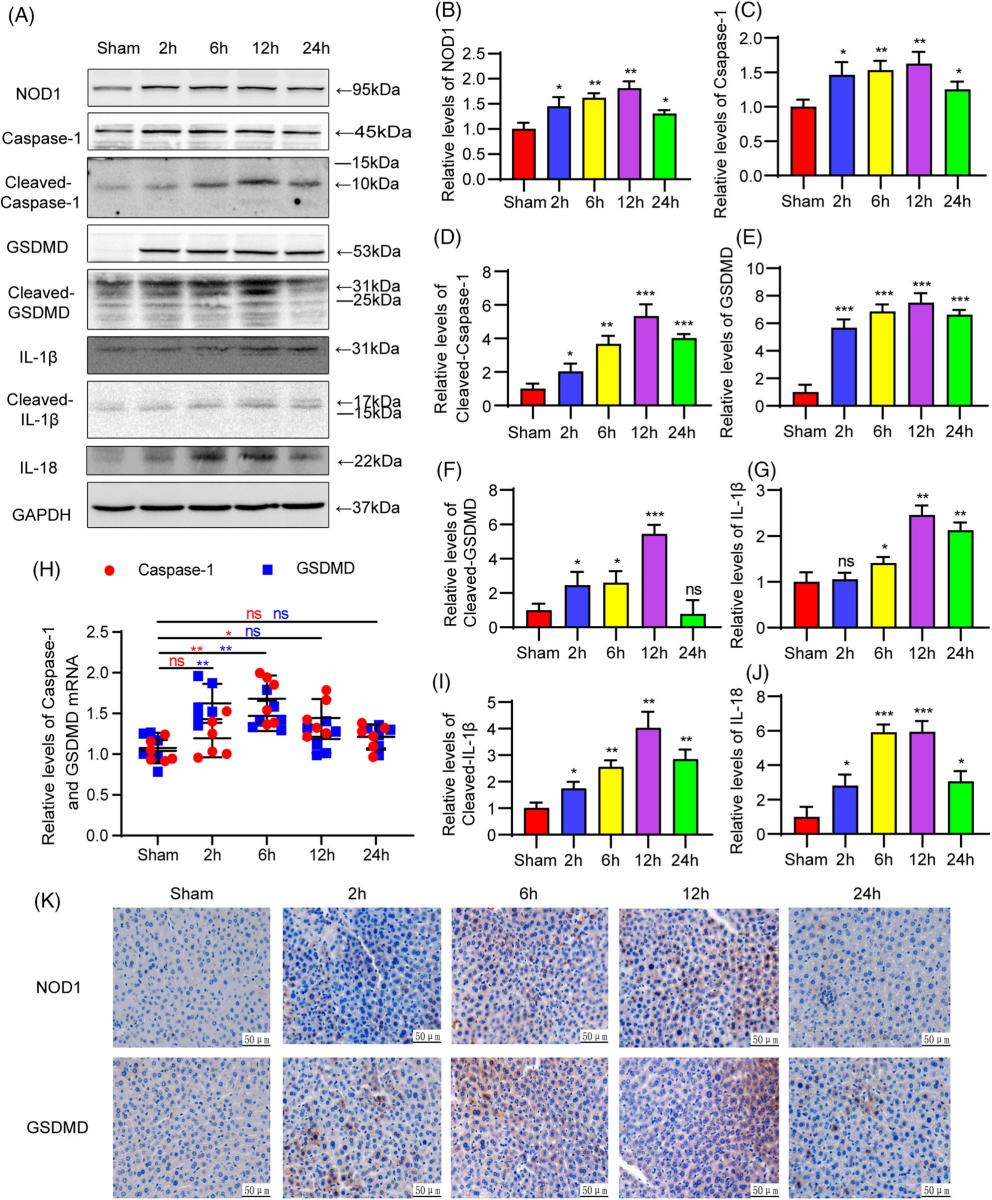
NOD1 and pyroptosis indicators in a mouse hepatic IRI model. (A) Western blot showing expressions of NOD1, Caspase‐1, cleaved‐Caspase‐1, and GSDMD cleaved‐GSDMD. IL‐13, cleaved‐IL‐1B, and IL‐18 protein levels were increased after IR treatment, achieving peak values at 6 or 12 h and then gradually decreasing (B–G and I and J). Statistical analyses of NOD1 and pyroptosis indicators in the different groups. (H) At each reperfusion time point in mouse liver samples subjected to IR, Caspase‐1, and GSDMD, mRNA were determined using qRT‐PCR analysis. (K) Expressions of NOD1 and GSDMD at each of the reperfusion time points as detected with use of immunohistochemistry. Compared with sham group: *p < 0.05, **p < 0.01, ***p < 0.001, **p < 0.0001

Following IR, NOD1 expression gradually increased, peaking at 12 h, and then decreasing, as based on results of western blots (Figures 2A, 2B, and 2K; *p* < 0.05). Caspase‐1 and IL‐1β protein contents were slightly increased, while either of their active forms, GSDMD and its active forms, or IL‐18 were all significantly increased as a function of reperfusion time, reaching highest levels at 6–12 h and decreasing thereafter (Figures 2A–2K; *p* < 0.05). Quantitative real‐time polymerase chain reaction (qRT‐PCR) results indicated that mRNA levels of Caspase‐1 peaked at 6 h and GSDMD at 2 h after reperfusion and then decreased (Figure 2H; Caspase‐1: IR6, 12 h vs. Sham; GSDMD: IR2, 6 h vs. Sham; *p* < 0.05). These results suggest that both the expression of NOD1 and pyroptotic death pathway proteins are involved in this mouse model of liver IRI.

### 2.3 iE‐DAP activates NOD1 and increases liver pyroptosis in a mouse model of liver IRI

**FIGURE 3:**
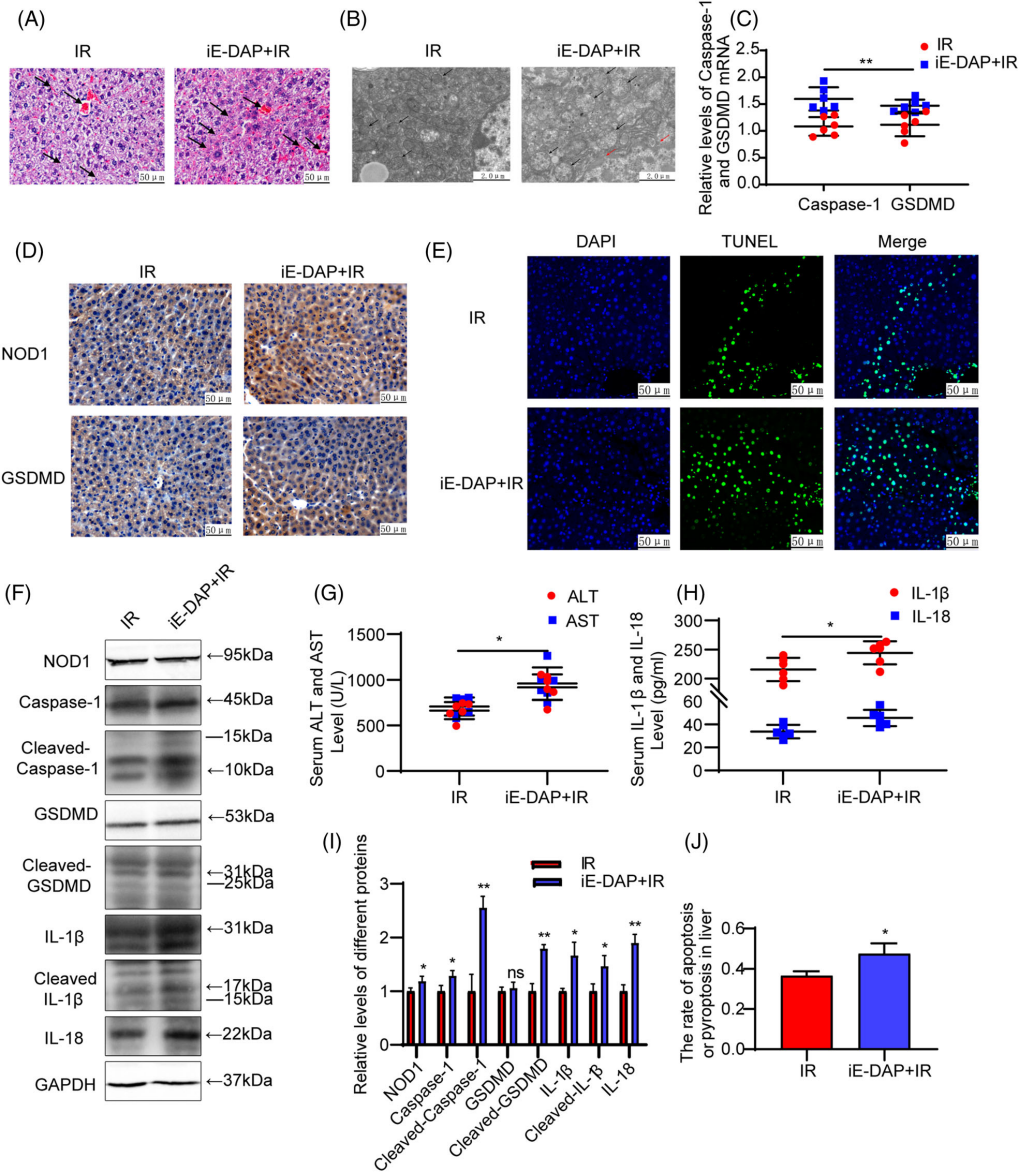
Activation of NOD1 by iE‐DAP on liver pyroptosis in a mouse hepatic IRI model. (A) HE staining was used to assess pathological changes in the liver of the IR and iE‐DAP + IR groups (×200, black arrow). (B) TEM images from the iE‐DAP + IR and IR groups. Compared with IR group. (C) mRNA levels of Caspase‐1 and GSDMD in mouse liver samples as measured by qRT‐PCR in the iE‐DAP + IR and IR groups. (D) Immunohistochemistry results displaying the expressions of NOD1 and GSDMD in mouse liver samples in the two groups. (E and J) Cell apoptosis or pyroptosis as detected using TUNEL, with representative images for the two groups. The apoptotic or pyroptotic nuclei were dyed green and normal nuclei blue. Rate of TUNEL‐positive cells in mouse liver samples were analyzed using lmageJ software. (F and I) Western blots demonstrating that expressions of NOD1, Caspase‐1, cleaved‐Caspase‐1, cleaved GSDMD, IL‐1B cleaved‐IL‐1β, and IL‐18 were increased in the iE‐DAP + IR versus the IR group. Statistical analysis of NODi and pyroptosis protein indicators in the iE DAP + IR and IR groups. (G and H) Serum levels of ALT and AST IL‐1β and IL‐18 in the iE‐DAP + IR group were increased as compared with the IR group: *p < 0.05, **p < 0.01, ***p < 0.001, ****p < 0.0001

Treatment with iE‐DAP increased hepatocyte pyroptosis and hepatic sinusoidal congestion in our mouse model of IRI (Figure 3A). There were significant increases in serum levels of AST and ALT in the iE‐DAP + IR as compared with that in the IR group (Figure 3G; *p* < 0.05), and IL‐1β and IL‐18 levels were also significant increased (Figure 3H; *p* < 0.05) as based on results from ELISA. Positive rates of TUNEL and numbers of apoptotic and pyroptotic cells were found in the iE‐DAP + IR versus IR group (Figures 3E and 3J; *p* < 0.05). As compared with that in the IR group, mRNA contents of Caspase‐1 and GSDMD were slightly increased in the iE‐DAP + IR group (Figure 3C; *p* < 0.01). Although protein expression levels of NOD1, Caspase‐1, and GSDMD were not significantly altered by iE‐DAP treatment, significant increases were obtained in expressions of their active forms as well as in IL‐1β, cleaved‐IL‐1β, and IL‐18 (Figures 3D, 3F, and 3I; *p* < 0.05). Results from TEM indicated that activation of NOD1 augmented morphological changes in cells during pyroptotic cell death, as there were significant increases in the number of hepatocyte membrane pores and swollen mitochondria within the iE‐DAP + IR group (Figure 3B). It appears that this capacity for NOD1 activation to trigger the pyroptosis signaling pathway affects cell morphology and protein expression, thereby aggravating liver IRI in mice

### 2.4 Effect of NOD1 downregulation on pyroptosis during IR in AML12 cells

**FIGURE 4:**
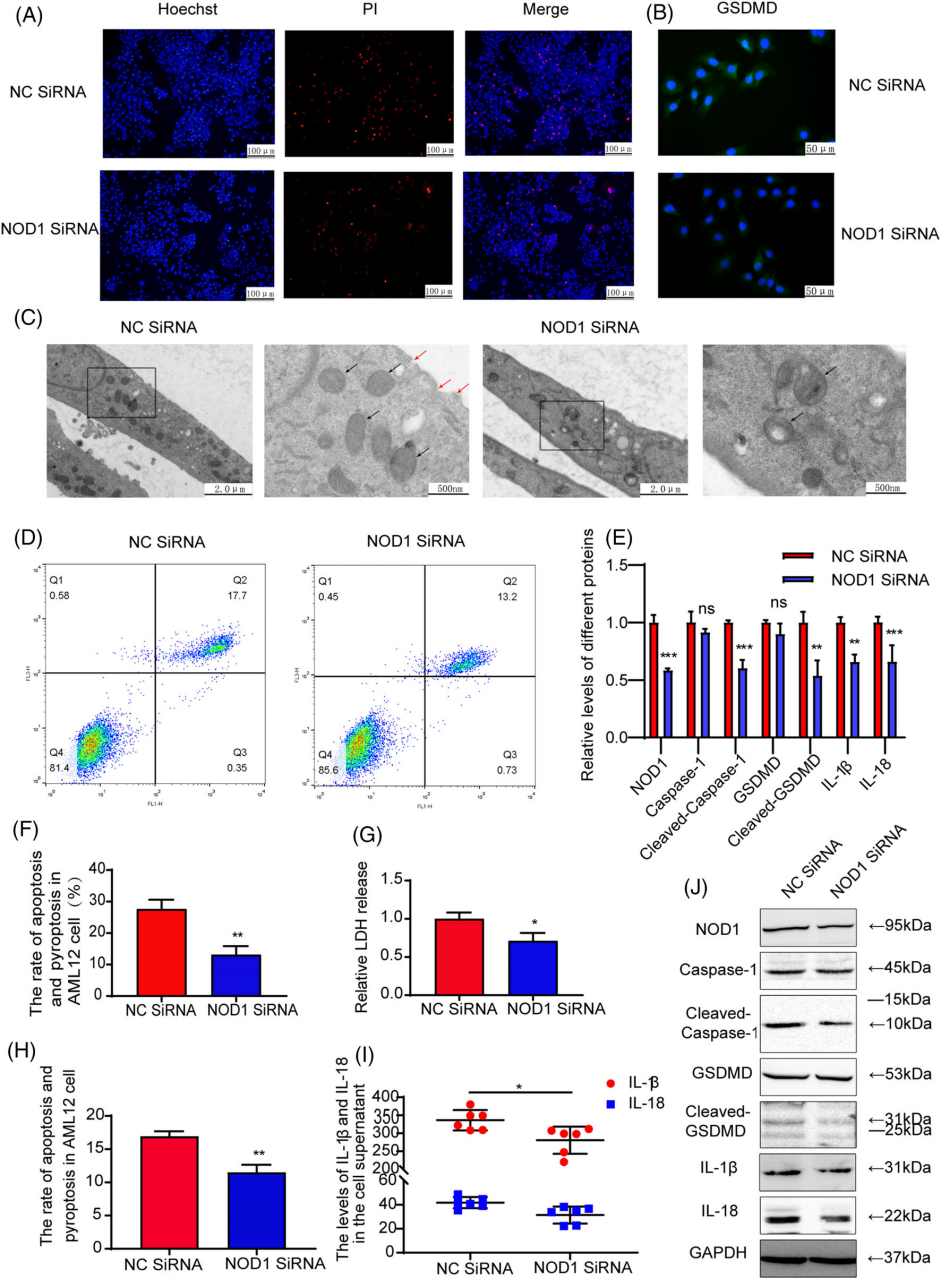
NOD1 knockout on pyroptosis in AML12 cells. (A and F) Hoechst/PI staining and positive rate of apoptosis or pyroptosis in AML12 cells in the NOD1 SiRNA and NC SiRNA groups. (B) Immunofluorescence of AML12 cells displayed a decrease in the intensity of the fluorescent signal within the GSDMD as compared with NC SiRNA group. (C) TEM images from the NC SiRNA and the NOD1 SiRNA groups. Compared with NC SIRNA. (D and H) Hepatocellular apoptosis or pyroptosis rates within the NC SiRNA and the NOD1 SiRNA groups. (E) Statistical analysis of NOD1 and pyroptosis protein indicators. (G) Relative release rate of LDH in AML12 cells. (I) Levels of IL‐18 and IL‐1β in the supernatant of AML12 cells in two groups. (J)Western blot analysis of NOD1, Caspase‐1, cleaved‐Caspase‐1, GSDMD cleaved‐GSDMD, IL‐1β, and IL‐18 in AML12 cells treated or not with NOD1 SiRNA: *p < 0.05, **p < 0.01, ***p < 0.001, ****p < 0.0001

NOD1 SiRNA pretreatment reduced protein expression levels of NOD1 after IR in AML12 cells. Although this decrease in NOD1 had little effect on Caspase‐1 and GSDMD expression levels, their active forms were significantly reduced (Figures 4B, 4E, and 4J; *p* < 0.05). In response to a decrease in NOD1, expression levels of IL‐18 and IL‐1β in AML12 cells (Figures 4E and 4J; *p* < 0.05) and their supernatants (Figure 4I; *p* < 0.05) were significantly decreased. Results of Hoechst/PI staining revealed that the number of bright blue and red (PI positive) as well as apoptotic and pyroptotic cells were decreased, while cell damage was alleviated in the NOD1 SiRNA group (Figures 4A and 4F; *p* < 0.01). ELISA results revealed that there was an approximately 20% reduction in LDH release (Figure 4G; *p* < 0.05) as well as a reduction of pyroptosis in these AML12 cells (Figures 4D and 4H; *p* < 0.01) within the NOD1 SiRNA as compared with that in the NC SiRNA group. TEM analysis showed that the number of pore formations and swollen mitochondria were decreased, while cell damage was reduced in the NOD1 SiRNA group (Figure 4C). These findings suggest that a downregulation of NOD1 attenuates the activation and release of inflammatory cytokines and cell morphologydamage during pyroptotic cell death in AML12 IR cells.

## 3 DISCUSSION

Pyroptosis represents a novel type of proinflammatory programmed cell death initiating from the recognition of DAMPs and PAMPs by the pattern recognition receptor. While pyroptosis is typically considered as an innate immune defense against intracellular bacteria, there is increasing evidence that it is also involved in aseptic inflammation, such as can occur with acute or chronic liver disease.^21^ Within the mouse model of liver IRI employed in this study the aseptic inflammation initiates innate and adaptive immunity. Our current results demonstrate that pyroptosis was related to processes involved with IRI in this mouse model of liver damage as revealed by the up‐regulation in expressions of cleaved‐Caspase‐1, cleaved‐GSDMD, and cleaved‐IL‐1β that occur at different time points following IR, as well as in the varying degrees of increased expressions of Caspase‐1, GSDMD, IL‐1β, and IL‐18. In addition, as compared with that in the sham group, serum levels of IL‐1β and IL‐18, number of TUNEL‐positive cells and cell membrane pores were all increased to varying degrees in response to IR.

NOD1 is widely expressed within hepatocytes and the liver.^22^ Following activation of NLRs, a series of reactions, including production of antibacterial factors and release of proinflammatory cytokines and chemokines produces cell death resulting from autophagy, apoptosis and pyroptosis.^16^, ^23^ Our goal in this report was to determine whether pyroptosis could be affected by the NOD1receptor. It has been established that activation of NOD2 can increase IL‐1β and IL‐18 in mouse eyes, and Caspase‐1 plays a crucial role in this process.^24^ However, NOD1 knockout has been demonstrated to significantly reduce the secretion of active IL‐1β in trophoblasts as induced by Chlamydia trachomatis.^25^ Cartwright et al.^26^ have reported that activation of NOD1 may lead to shock and multiple organ injury or dysfunction, while NOD1 inhibitors reduce the expression of adhesion molecules and alleviates hepatic IRI.^27^ Further support indicating damaging effects of NOD1 have been revealed from results demonstrating protective effects against hepatic IRI within NOD1 knockout mice.^28^

Given the above findings and results showing the increases in NOD1 and pyroptosis indicators that occur during hepatic IR in mice, we next wanted to examine some of the mechanisms of NOD1 activation on pyroptosis in this mouse model of liver IRI. One approach to address this issue involved treatment of mice with an intraperitoneal injection of the NOD1 agonist, iE‐DAP, prior to liver IR. This iE‐DAP treatment not only increased the expression of NOD1, but also expressions of the full‐length and active cleavage fragments of pyroptosis. Positive rates of TUNEL and levels of IL‐1β and IL‐18 increased lightly with the activation of NOD1 during IRI, as achieved in the iE‐DAP + IR group. In addition, when assessing cell morphology there were clear increases in the number of pore formations in response to this activation of NOD1. These results demonstrate that NOD1 activation increases pyroptosis and damages hepatocytes during IRI. To further assess these effects of NOD1 on pyroptosis, we examined the effects of downregulating NOD1 expression prior to IR in AML12 cells. After downregulating the expression of NOD1, the GSDMD signal, active forms of Caspase‐1 and GSDMD and expressions of IL‐1β and IL‐18 were all decreased in these AML12 cells. In addition, contents of IL‐1β and IL‐18 in the supernatant, rate of PI positive cells and relative release of LDH were also decreased with the inhibition of NOD1 expression. Results from the flow cytometry assay revealed that Annexin v/PI double positive cells were decreased following the downregulation of NOD1 expression, while TEM images showed that the number of cell and nuclear membrane ruptures were decreased under these conditions. Accordingly, with a reduction in NOD1 expression cellular and nuclear membrane damage during IR is decreased, as is the release of LDH and the inflammatory factors, IL‐1β and IL‐18, thus affecting pyroptosis within AML12 cells. When collating results from previous literature as described above and our current experimental findings, it appears that NOD1 may directly affect pyroptosis by recruiting caspase‐1. Such an effect may then incorporate a number of potential events including: (1) promotion of IL‐1β and IL‐18 release, (2) aggravation of inflammatory reactions and hepatocyte injury, (3) activation of the NF‐KB pathway through Caspase‐1 recruitment, and/or (4) activation of pyroptosis through other pathways such as PI3K/Akt and MAPK. The specific pathway involved with pyroptosis remains to be determined.

In conclusion, our current results demonstrate that pyroptosis is involved in liver IRI. Activation of NOD1 enhances pyroptosis in mouse hepatic IRI, while a downregulation of NOD1 expression reduces pyroptosis and damage in AML12 cells in response to IR. Based on these findings, we conclude that NOD1 plays an important role in liver IRI. The identification of this relationship between NOD1 and pyroptosis serves as an important foundation for future work directed toward the development of novel treatments for liver IRI.

## 4 MATERIALS AND METHODS

### 4.1 Animals and cell lines

Forty two male C57BL/6 mice (Huafukang, Beijing, China; *n* = 6/group), 8–10 weeks old, were maintained in specific‐pathogen‐free environment at approximately 23°C. Mouse experiments were performed in the Laboratory Animal Center of the Institute of Radiation Medicine, Chinese Academy of Medical Sciences and were conducted in adherence to the Guidelines for the Management and Use of Laboratory Animals. The protocol was approved by the Ethics Committee for Animal Experiments of the Institute of Radiation Medicine, Chinese Academy of Medical Science. The alpha mouse liver 12 (AML12) cell line was purchased from the Chinese Academy of Sciences (Shanghai, China). All experiments were replicated a minimum of three times.

### 4.2 Reagents and antibodies

Dulbecco's modified Eagle's medium/F12 medium and fetal bovine serum were purchased from Gibco (ThermoFisher Scientific, Shanghai, China). The antibodies NOD1 and cleaved‐GSDMD were from Cell Signaling Technology Inc. USA; IL‐1β, IL‐18, and Caspase‐1 were from the Abcam Company, UK) and GSDMD and GAPDH were purchased from Abclonal (Wuhan, China). The cell hypoxia device was purchased from the Billups‐Rothenber Company (USA).

### 4.3 Animal model

Mice were food restricted but had free access to water 8 h prior to treatment. Following anesthesia with 1% pentobarbital sodium (50 mg/kg, ip), a segmental (70%) warm ischemia procedure was performed by blocking the left lateral and median lobes with a vascular clamp. After 60 min, the clamp was removed, followed by reperfusion at 2, 6, 12, or 24 h. Mice were then euthanized by cervical dislocation at 2, 6, 12, or 24 h after reperfusion. For the sham group, only the first hepatic portal vein was exposed. In order to assess the impact of NOD1 in pyroptosis during IR in this model, mice received an intraperitoneal injection of γ‐d‐glutamyl‐meso‐diaminopimelicacid (iE‐DAP; 5000 μg/kg; invivoGen, USA)^19^, ^29^ prior to ischemia. The IR control group was treated with an injection of an equal amount of normal saline.

### 4.4 Cell culture and treatment

AML12 cells were inoculated in six‐well plates with 2.5 × 10^5^ cells per well and divided into two groups. For the NC small interfering RNA (SiRNA; RiboBio, Guangzhou, China) group, cells were transfected with 50 nmol/L NC SiRNA for 36 h before ischemia, then reperfused for 12 h. For the NOD1 SiRNA (RiboBio) group, cells were transfected with 50 nmol/L NOD1 SiRNA for 36 h before ischemia, then reperfused for 12 h.

### 4.5 Serology detection

After the conclusion of the liver IR, 1 ml of blood was obtained from the inferior vena cava. The blood sample was kept at room temperature for 60 min, then centrifuged at 1420 g  for 15 min and the serum was collected. An automatic biochemical instrument for use in animals (Mindray BS‐240VET) was used for determining alanine aminotransferase (ALT) and aspartate aminotransferase (AST) in these mouse serum samples.

### 4.6 Enzyme‐linked immunosorbent assay

Cytokine levels (IL‐18 and IL‐1β) in mouse serum and supernatants of AML12 cells were detected with use of enzyme‐linked immunosorbent assay (ELISA) kits (MultiSciences, Beijing, China) according to the instructions provided with the kit.

### 4.7 Liver histopathology

Liver parenchymas were fixed with 4% paraformaldehyde, dehydrated, and embedded in paraffin. Sections (4‐μm) from paraffin‐embedded tissues were then prepared for hematoxylin and eosin (HE) staining.

### 4.8 Immunohistochemistry

Liver paraffin sections (4 μm) were dewaxed and hydrated. After antigen retrieval with sodium citrate, the samples were incubated in 3% H_2_O_2_ to determine endogenous peroxidase activity, then incubated with NOD1 and GSDMD antibody (1:200) at 4°C overnight. On the following day, the secondary antibody (Cell Signaling Technology, USA) was added for 1 h and then visualized using diaminobenzidine.

### 4.9 Immunofluorescence

AML12 cells were transfected following IR in 24‐well plates, fixed at −20°C with precooled anhydrous methanol for 15 min and then incubated in 1% triton X‐100 for 15 min. The GSDMD antibody (1:200) was then used for incubation with AML12 cells at 4°C overnight. The second antibody was administered for 1 h in the dark on the second day. 2‐(4‐Amidinophenyl)‐6‐indolecarbamidine dihydrochloride staining was then used to observe the results.

### 4.10 Transmission electron microscopy

Liver tissue was fixed in 2.5% glutaraldehyde electron microscope fixative solution for 2 h, washed with phosphate‐buffered saline (PBS), and then postfixed with 2% osmium tetroxide. The tissue was then dehydrated, embedded, sliced, and stained for transmission electron microscopy (TEM) observation.

### 4.11 TdT‐mediated dUTP nick‐end labeling assay

Terminal deoxynucleotidyl transferase nick‐end labeling (TUNEL) kits (Roche, Switzerland) were used according to the manufactures’ instructions. The PBS solution containing 1% H_2_O_2_ was added to the paraffin sections and washed. The TUNEL mixed working solution (liquid A:liquid B = 9:1) was then added, incubated in a wet box for 60 min and paraffin sections were then sealed. The total number of TUNEL‐positive cells and hepatocytes were observed under light microscopy.

### 4.12 Hoechst/propidium iodide staining

Pore formations in cell membranes were observed with use of a cell apoptosis and necrosis detection kit (Beyotime, Shanghai, China). Hoechst and propidium iodide (PI) were added to the treated cells under dark conditions and incubated at 4°C for 20 min. Cells were washed three times with PBS and the resultant red and blue cells were observed under fluorescent microscopy.

### 4.13 Flow cytometry assay

Cells in the NOD1 SiRNA and NC SiRNA groups (approximately 1 × 10^6^ cells in each group) were washed with cold PBS and suspended in 1 ml binding buffer. Cells (100 μl) were added into each tube, followed by an administration of 5 μl annexin V‐fluorescein isothiocyanate and PI solution into each tube. Cells were then incubated for 10 min at room temperature in the dark. Each specimen was analyzed using FACS Calibur flow cytometry (BD Biosciences, San Jose, CA).

### 4.14 Relative LDH release

Treated cells were centrifuged at 89 g for 2 min, and relative release of LDH was detected with use of ELISA in accordance with the instructions of the LDH cytotoxicity test kit (BioLigend, USA).

### 4.15 Western blot analysis

AML12 cells and liver tissue samples were fully ground in RIPA (Solarbio, Beijing, China) lysate. After centrifugation at 12787 g for 20 min, the supernatant was collected, followed by protein quantification with the use of the bicinchoninic acid (Solarbio) protein detection kit. Proteins with identical concentrations and volumes from each sample were run on different (10% and 12%) sodium dodecylsulfate‐polyacrylamide gels. The proteins were transferred to polyvinylidene fluoride (PVDF; Millipore, MA, USA) membranes, sealed in 5% skimmed milk for 1.5 h and probed using the following antibodies: NOD1, Caspase‐1, cleaved‐Caspase‐1, GSDMD, cleaved‐GSDMD, IL‐1β, cleaved‐IL‐1β, IL‐18, and GAPDH at 4°C overnight. They were then incubated with the secondary antibody for 1.5 h. An equal volume of chemiluminescence solution was mixed with the PVDF membranes and exposed in G: box machine.

### 4.16 Quantitative real‐time polymerase chain reaction

Total RNA was obtained from mouse liver samples using RNATRIZOL (TransGen Biotech, Beijing, China). Total cDNA was synthesized in a 20 μl system using TransScript First‐Strand cDNA Synthesis SuperMix (TransGen Biotech). According to directions of the TransStart Top Green qPCR SuperMix kit (TransGen Biotech), 0.1 μg cDNA was amplified continuously in a 20 μl polymerase chain reaction (PCR) reaction system. The following primers were used in this study: GSDMD (F: 5′‐ATGCCATCGGCCTTTGAGAAA′, R:5′‐AGGCTGTCCACCGGAATGA‐3′), GAPDH (F:5′‐CCCCCAATGTATCCGTTGTG‐3′, R:5′‐TAGCCCAGGATGCCCTTTAGT‐3′), Caspase‐1 (F:5′‐ACACGTCTT‐GCCCTCATTATCT‐3′, R:5′‐ATAACCTTGGGCTT‐GTCTTTCA‐3′).

### 4.17 Data analysis

The data are presented as means ± SDs. Student's *t*‐tests were used for comparisons involving two groups and one‐way ANOVAs for multiple groups. The software PRISM 8.0.2 (GraphPad Software Inc., San Diego, CA, USA) was used for these statistical analyses. Each experiment was replicated a minimum of three times. A *p* < 0.05 was required for results to be considered as statistically significant. *p < 0.05, **p < 0.01, ***p < 0.001, ****p < 0.0001.

## CONFLICT OF INTERESTS

The authors declare that there are no competing interests associated with the manuscript.

## ETHICS STATEMENT

Experiments were performed under a project license (NO.: IRM‐DWLL‐2020092) granted by the committee on the Ethics of Animal Experiments of the Institute of Radiation Medicine, Chinese Academy of Medical Sciences, in compliance with the Guidelines for the Management and Use of Laboratory Animals.

## AUTHOR CONTRIBUTIONS

Jinzhen Cai and Guoliang Zhang designed the study. Yu Liu carried out the experiments and prepared the manuscript. Shipeng Li analyzed the results. All authors contributed to revising the manuscript and reviewing the final draft.

## FUNDING

The present study was supported by the National Natural Science Foundation of China [grant number 81670600].

## Data Availability

Experimental data related to the article are available from the corresponding author.
